# Human leukocyte antigen-G expression in differentiated human airway epithelial cells: lack of modulation by Th2-associated cytokines

**DOI:** 10.1186/1465-9921-14-4

**Published:** 2013-01-18

**Authors:** Steven R White, Dagan A Loisel, Randi Stern, Bharathi Laxman, Timothy Floreth, Bertha A Marroquin

**Affiliations:** 1 University of Chicago, Section of Pulmonary and Critical Care Medicine, 5841 S. Maryland Ave, MC 6076, Chicago, IL 60637, USA; 2 From the Departments of Medicine and Human Genetics, Chicago, IL 60637, USA; 3 Human Genetics, University of Chicago, Chicago, IL 60637, USA

**Keywords:** HLA-G, Airway epithelium, IL-13, IL-4, IL-5, IL-10, Asthma

## Abstract

**Background:**

Human leukocyte antigen (HLA)-G is a nonclassical class I antigen with immunomodulatory roles including up-regulation of suppressor T regulatory lymphocytes. HLA-G was recently identified as an asthma susceptibility gene, and expression of a soluble isoform, HLA-G5, has been demonstrated in human airway epithelium. Increased presence of HLA-G5 has been demonstrated in bronchoalveolar lavage fluid recovered from patients with mild asthma; this suggests a role for this isoform in modulating airway inflammation though the mechanisms by which this occurs is unclear. Airway inflammation associated with Th2 cytokines such as IL-4 and IL-13 is a principal feature of asthma, but whether these cytokines elicit expression of HLA-G is not known.

**Methods:**

We examined gene and protein expression of both soluble (G5) and membrane-bound (G1) HLA-G isoforms in primary differentiated human airway epithelial cells collected from normal lungs and grown in air-liquid interface culture. Cells were treated with up to 10 ng/ml of either IL-4, IL-5, or IL-13, or 100 ng/ml of the immunomodulatory cytokine IL-10, or 10,000 U/ml of the Th1-associated cytokine interferon-beta, for 24 hr, after which RNA was isolated for evaluation by quantitative PCR and protein was collected for Western blot analysis.

**Results:**

HLA-G5 but not G1 was present in dAEC as demonstrated by quantitative PCR, western blot and confocal microscopy. Neither G5 nor G1 expression was increased by the Th2-associated cytokines IL-4, IL-5 or IL-13 over 24 hr, nor after treatment with IL-10, but was increased 4.5 ± 1.4 fold after treatment with 10,000 U/ml interferon-beta.

**Conclusions:**

These data demonstrate the constitutive expression of a T lymphocyte regulatory molecule in differentiated human airway epithelial cells that is not modulated by Th2-associated cytokines.

## Introduction

Asthma is one of the most common chronic medical disorders in the Western world with an increasing prevalence and significant morbidity [1,2]. T lymphocytes commonly infiltrate into the mucosa of asthmatic airways [3,4], and environmental exposures may trigger an “over-zealous” response of airway-resident T helper 2 (Th2)-subclass CD4+ lymphocytes [3-6]. Fluid recovered by bronchoalveolar lavage from asthmatic airways is enriched in interleukin (IL)-4, IL-5, IL-13 and granulocyte-macrophage colony stimulating factor (GM-CSF) but not IFN-γ [5], indicating the presence of Th2 subclass CD4+ lymphocytes. Epithelial cells stimulated with either IL-4 or IL-13 express cytokines and chemokines that trigger and maintain airway inflammation [7-11]. Both IL-4 and IL-13 elicit changes in epithelial structure, morphology and differentiation that contribute to both airway inflammation and remodeling [12-14].

Human leukocyte antigen-G (HLA-G) is a non-classical, class Ib, major histocompatibility complex antigen [15]. HLA-G differs from classical Ia molecules because of its limited polymorphism in the coding region and a somewhat restricted tissue distribution. Alternative splicing results in transmembrane (G1) and soluble (G5) isoforms that dimerize with β2-microglobulin (β2m) light chains similar to other class I molecules [16,17].

Originally considered a pregnancy-specific HLA with a critical role in maintaining immune tolerance toward the allogenic fetus [18-20], it is now appreciated that HLA-G is expressed in adult tissues [21]. Recent studies in asthmatic families and in a birth cohort at high risk for developing asthma suggest a role for HLA-G in asthma susceptibility [22,23]. Associations in genetic variations in the promoter region [22] and in a microRNA target site in the 3’UTR [23] of HLA-G suggested that dysregulated expression may contribute to asthma pathogenesis [22,23]. A soluble protein isoform of HLA-G, HLA-G5, is present in airway epithelial cells in vivo [22]. Increased circulating levels of G5 was reported in two studies of children with atopic asthma [24,25], and G5 was present in greater abundance in bronchoalveolar lavage fluid collected from subjects with mild, persistent asthma compared to control subjects [26].

HLA-G is a ligand for the leukocyte Ig-like receptor (LILR) B1 (also referred to as inhibitory receptor Ig-like transcript (ILT)2, CD85j), expressed by human NK cells, monocytes, T cells, B cells and dendritic cells [27], and the myeloid-specific LILRB2 (ILT4, CD85d) receptor [28] with high specificity [29]. In vitro data indicate that HLA-G inhibits both NK cell and CD8+ T cell mediated cytolysis [30], suppresses CD4+ T cell alloproliferative responses [31], and induces apoptosis of CD8+ T cells [32]. Moreover, HLA-G can down-regulate the expression of CD4 and CD8 on allostimulated T cells [33], and promote maternal immune cell cytokine release toward a Th2-skewed profile [34]. HLA-G then may be an attractive candidate molecule for modulating specific T cell profiles that are important in asthma. Further, HLA-G production may in turn be regulated by the pleiotropic, immunoregulatory cytokine IL-10 which may in the context of asthma further regulate T cell profiles and function. IL-10 is generally a suppressive cytokine that inhibits inflammatory mediator expression and suppresses selected T cell activation pathways. The production of both IL-10 and sHLA-G in peripheral blood mononuclear cells (PBMCs) collected from subjects with asthma and stimulated with lipopolysaccharide (LPS) is lower than that seen in similarly-treated cells collected from normal subjects [35], and addition of exogenous IL-10 to asthmatic PBMCs restored sHLA-G production to concentrations seen in normal PBMCs after LPS stimulation [36].

Given the multiple effects of Th2-associated cytokines on airway inflammation [7-11] and remodeling [12-14], it was unclear whether IL-4 and IL-13 would stimulate HLA-G expression and abundance in airway epithelial cells that would be counter-regulatory to inflammation, or whether, as promoters of airway inflammation, IL-4 and IL-13 would actually downregulate HLA-G expression. Further, IL-10 and the IL-10 promoter region [37] contain polymorphisms that are associated with asthma susceptibility, though this is disputed in other studies [38], and serum IL-10 concentrations are lower in children with atopic asthma compared to normal children [25]. Thus decreased production of IL-10 in asthma might in turn lead to changes in HLA-G expression. To test these hypotheses, we examined HLA-G expression and abundance in a culture model of differentiated airway epithelial cells (dAEC) that has proven to be useful in examining epithelial cell function. We then examined whether the Th2-associated cytokines IL-4, IL-5 or IL-13, the Th1-associated interferon-beta (IFN-β), or the immunoregulatory cytokine IL-10 regulated HLA-G expression. We demonstrate the constitutive expression of soluble G5 in differentiated AEC, and that IFN-β but not IL-10 nor any Th2-associated cytokine regulates expression of G5.

## Materials and methods

### Materials

Antibodies directed against HLA-G (4H84 clone), HLA-G5 (5A6G7 and MEM-G/9) or HLA-G1 (01 G) were obtained from Exbio, Inc. (Prague, Czech Republic). Antibodies directed against β-tubulin were obtained from Abcam, Inc. (Cambridge, MA). Antibodies directed against IL-10R1 and IL-10R2 were obtained from R&D Systems, Inc. (Minneapolis, MN). Antibodies directed against IL-10R1, cytokeratin-5 (CK5) and MUC5AC were obtained from Santa Cruz Biotechnologies, Inc. (Santa Cruz, CA). All secondary antibodies were obtained from Invitrogen, Inc. (Carlsbad, CA). All other reagents were obtained from Sigma-Aldrich, Inc. (St. Louis, MO).

### Cell culture

We have described previously methods for collection of primary human airway epithelial cells from donated lungs and their culture in air-liquid interface (ALI) for 3 wk to generate dAEC [39]. These cells develop characteristics of differentiated cells with appearance of cilia, secretion of mucins, and the presence of markers such as β-tubulin (for ciliated cells) and MUC5AC (for goblet cells) [39,40]. Cells were collected from lungs provided by the Regional Organ Bank of Illinois (Elmhurst, IL).

### Confocal microscopy

We have described this method previously [39,40]. Cells were stained with two primary antibodies. The first was directed against either cytokeratin 5 (CK5) (mouse anti-human, sc-32721, or goat anti-human, sc-17090) to mark basal cells [41,42], MUC5AC (rabbit anti-human, sc-20118, or goat-anti-human, sc-16910) to mark goblet cells [43,44], or β-tubulin (rabbit anti-human, ab6046, or goat anti-human, ab21057) to mark ciliated cells [45]. The second was directed against soluble G5 (MEM-G/9 or 5A6G7 mAbs). Background was calculated from images collected after staining that omitted the primary antibody; this was subtracted from all images using ImageJ (Wayne Rasband, National Institutes of Health, Bethesda, MD). For each z-stack generated, x,y slices were selected starting from the membrane on which cells were grown, and progressing to the top of the cells; these slices then were combined using a maximum intensity protocol (ImageJ) to generate final images. At least three separate experiments were done for each set of antibodies used, and representative images were selected.

### Quantitative real-time polymerase chain reaction (qPCR)

Total RNA was isolated from cells using TRI Reagent (Sigma) following the manufacturer’s protocol. Samples were treated with DNase I (Ambion, Austin, TX). Total RNA was reverse transcribed using random primers and Superscript II reverse transcriptase (Invitrogen, Carlsbad, CA). qPCR was performed using Platinum SYBR Green qPCR SuperMix-UDG with ROX (Invitrogen). Either primers to amplify all HLA-G isoforms (pan-G, Forward: 5’-CTGACCGAGACCTGGGCGGGCT-3’; Reverse: 5’-GGCTCCATCCTCGGACACGCCGA-3’), or isoform-specific primers to amplify either HLA-G5 (Forward: 5’-TGCTGCAGCGCGCGGA-;3’;Reverse: 5’-GGCCTCA CCACCGACCCTGTT-3’) or HLA-G1 (Forward: 5’-GC TGCAGCGCGCGGAC-3’; Reverse: 5’-TGGTGGGCAG GGAAGACTGCTT-3’) were used. cDNA samples were tested in triplicate and normalized to 18S-sRNA expression, calculated using comparative cycle-time ratios. In some experiments the JEG-3 choriocarcinoma cell line was used as a positive control for HLA-G expression.

### Western blot analysis

Densitometry was done using ImageJ software for both HLA-G and β-actin blots; the ratio of HLA-G blot density to β-actin blot density reflected the relative abundance in each lysate, as previously described [40,46].

### Data analysis

Data are expressed as mean ± SEM. Differences were examined by analysis of variance; when significant differences were found, post-hoc analysis was done using Fisher’s protected least significant difference test. Differences were considered significant when P < 0.05.

## Results

### HLA-G is expressed in dAEC

**Figure 1:**
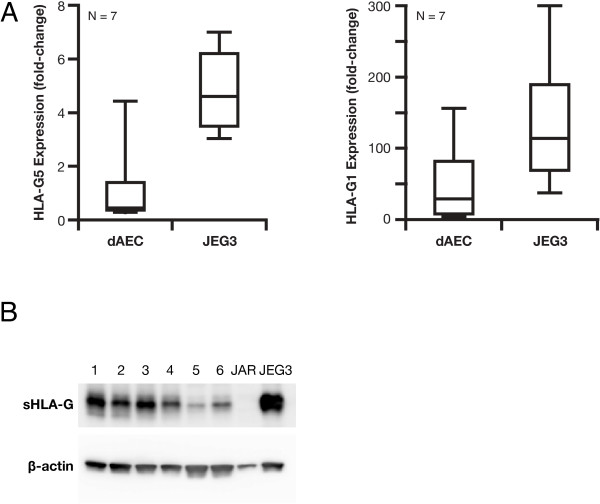
**Demonstration of HLA-G in primary dAEC grown in ALI culture. A**. Expression of HLA-G as demonstrated by qPCR. One sample from each subject was tested in triplicate and normalized to 18S-RNA expression. A positive control cell line (JEG-3) analyzed at the same time is shown for reference. **B**. Protein expression of HLA-G as demonstrated by western blot. Whole cell lysates collected from six different dAEC cultures were resolved using the 4H84 mAb that binds both, and cannot distinguish between, the soluble G5 and membrane bound G1 isoforms. A single band at ~ 39 kD consistent with HLA-G is demonstrated. A negative control, the JAR choriocarcinoma cell line that does not express HLA-G, and a positive control, the JEG3 choriocarcinoma cell line, are shown.

We first examined the expression of HLA-G in primary dAEC grown in ALI culture. RNA collected from dAEC grown in ALI culture × 3 wk was examined for the expression of G1 and G5 using qPCR. Both isoforms were expressed in dAEC (Figure 1A). We then examined whether gene expression translated into protein expression. Whole cell lysates collected from dAEC grown in ALI culture × 3 wk were resolved by Western blot. Using the 4H84 mAb that recognizes the soluble G5 and a shed G1 HLA-G, a single band at ~39 kD consistent with sHLA-G was demonstrated (Figure 1B). The presence of G1 protein could not be ascertained separately by western blot as the only antibody specific for this isoform (01 G) was not suitable for western blot analysis in preliminary experiments (data not shown). These data demonstrated both G1 and G5 gene expression and significant sHLA-G protein expression in dAEC.

**Figure 2:**
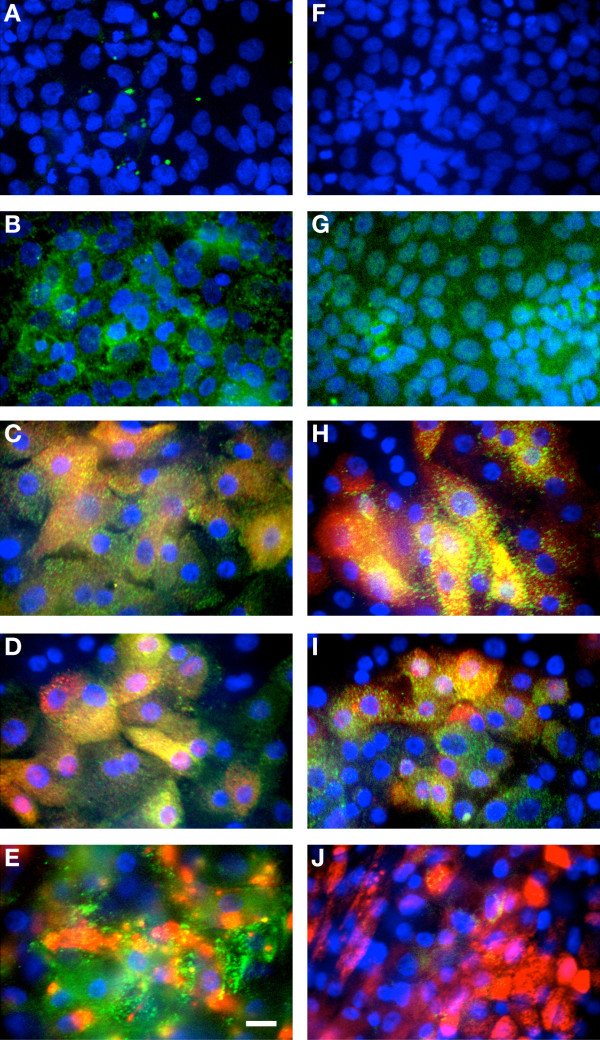
**Presence of soluble HLA-G5 and membrane-bound HLA-G1 in dAEC by confocal microscopy.** Cells were labeled using the 5A6G7 antibody for G5 (**A** - **E**) or the 01G antibody for G1 (**F** - **J**) (green in each image), and nuclei were counter-stained with Hoechst 33258 (blue). The negative control cell line JAR (**A**,**F**) and positive control cell line JEG-3 (**B**,**G**) are shown. Epithelial cells were also labeled for cytokeratin 5 (**C**,**H**), MUC5AC (**D**,**I**) or β-tubulin (**E**,**J**) (red in each image). Yellow label represents the overlap of both G5/G1 and the cell subtype marker. Background control fluorescence for each color in each image was subtracted. Images representative of 3 experiments. Bar in (**E**), 10 μm for all images.

To characterize the localization of G5 by confocal microscopy we used the 5A6G7 mAb, which targets epitopes in the translated intron 4 sequence of this soluble isoform, to label dAEC followed by confocal microscopy. The choriocarcinoma cell lines JAR and JEG3 were used as negative and positive controls, respectively. G5 was localized to basal and goblet cells of dAEC, whereas ciliated columnar cells had somewhat less abundance (Figure 2A-E). We then examined whether membrane-bound G1 was present using the 01 G mAb. Modest G1 localization could be demonstrated in both basal and goblet cells, and none in ciliated columnar cells (Figure 2F-J).

### Effect of Th2-associated cytokines on HLA-G gene and protein expression

**Figure 3:**
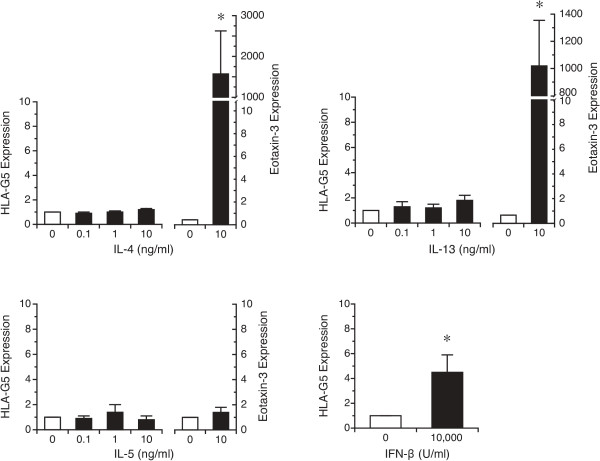
**Expression of HLA-G after treatment with IL-4, IL-5 or IL-13 treatment as demonstrated by qPCR.** dAEC were treated with 0.1 - 10 ng/ml of each cytokine, or 10,000 U/ml of IFN-β as a positive control, for 24 hr, after which RNA expression was examined by qPCR using G5-specific primers. For experiments in which cells were treated with a Th2-associated cytokine, expression of eotaxin-3 was measured as a positive control of response. N = 6 experiments each for IL-13 and IL-4, 4 experiments for IL-5 and 8 experiments for IFN-β. *, P < 0.05 vs no treatment.

**Figure 4:**
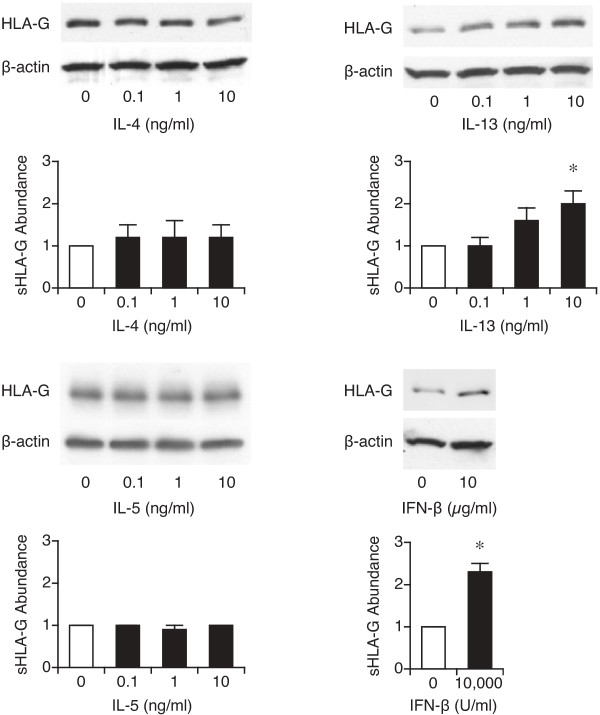
**Protein expression of HLA-G after treatment with IL-4, IL-5 or IL-13 treatment as demonstrated by western blot.** dAEC were treated with 0.1 - 10 ng/ml of each cytokine, or 10,000 U/ml of IFN-β as a positive control, for 24 hr, after which whole cell lysates were resolved using the 4H84 antibody. Densitometry was done for each blot and normalized to control. N = 5 experiments each for IL-13 and IL-5, and 4 experiments each for IL-4 and IFN-β. *, P < 0.05 vs no treatment.

We first examined whether treatment of dAEC with IL-4, IL-5 or IL-13 would elicit HLA-G5 expression. Cells were treated with 0.1 - 10 ng/ml each cytokine, or 10,000 U/ml IFN-β as a positive control [47,48], for 24 hr. There was no clear concentration-response to any of the Th2-associated cytokines using either the G5 specific (Figure 3) or pan-G primers (data not shown), though IFN-β treatment elicited significant HLA-G5 expression, and both IL-4 and IL-13 increased eotaxin-3 expression [49,50], as expected. Similarly, HLA-G protein expression did not increase significantly following treatment with IL-4 over 24 hr (Figure 4). Treatment with IL-13 elicited increased protein expression at the highest concentration used, 10 ng/ml (Figure 4), but this was not matched by a change in G5 gene expression for the same concentration of IL-13 (Figure 3). These data suggested that no Th2-associated cytokine increased expression of the constitutively-expressed HLA-G.

### Effect of IL-10 on HLA-G gene and protein expression

**Figure 5:**
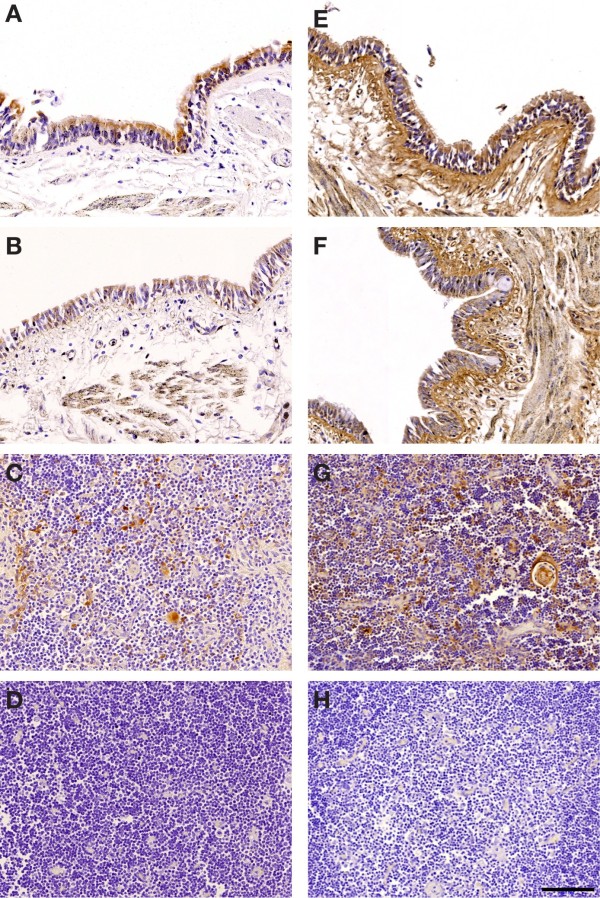
**Demonstration of IL-10 receptors in human airways obtained from two normal subjects (A, B, E, F), and normal human thymus (C, D, G, H) as a control, using the AF-274 antibody for IL-10R1 (A - D) and MAB874 antibody for IL-10R2 (E - H).** Representative of 3 experiments. Bar, 100 μm for all images.

**Figure 6:**
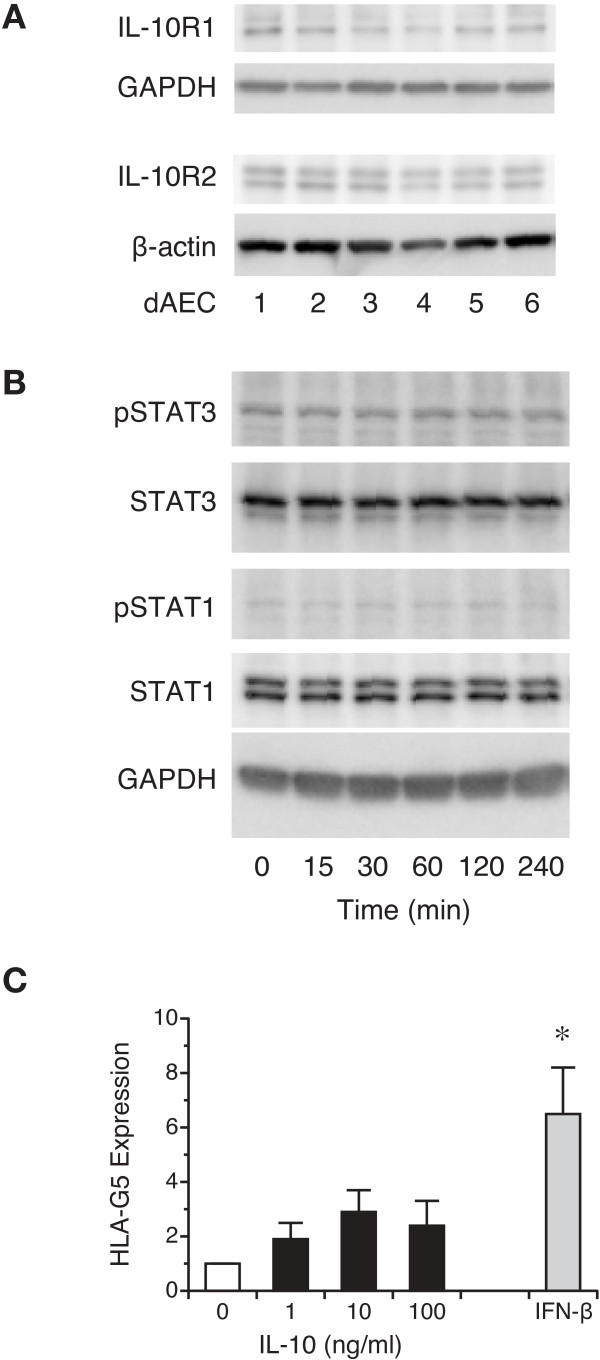
**A. Protein expression of six separate dAEC cultures resolved for IL-10R1 and IL-10R2.** Lane loading controls resolved for β-actin are shown. **B**. Phosphorylation of STAT3 in dAEC after treatment with 100 ng/ml IL-10 for up to 4 hr. Lane loading controls resolved for GAPDH are shown. Representative of 3 experiments. **C**. Expression of HLA-G after treatment with IL-10 treatment as demonstrated by qPCR. dAEC were treated with 1–100 ng/ml of IL-10, or 10,000 U/ml of IFN-β as a positive control, for 24 hr, after which RNA expression was examined by qPCR using G5-specific primers. N = 5 experiments. *, P < 0.02 vs no treatment.

There is controversy over the presence of IL-10 receptor subunits in airway epithelium: one study examined the presence of the IL-10R1 (also IL-10Rα) subunit, responsible for the specific binding of IL-10, in freshly-collected bronchial epithelial cells and in the BEAS-2B and 16HBE14o- cell lines, demonstrating the absence of this subunit [51]. The IL-10R2 (also IL-10Rβ), responsible for recruiting protein kinases of the Jak family and subsequent phosphorylation of the STAT1 and STAT3 transcription factors [52], is ubiquitously expressed and associated not only with IL-10R1 but with other receptor units such as IL-22R and IL-26. We first examined whether IL-10R1 and IL-10R2 were present in normal human airways. Using immunoperoxidase-based staining methods, we demonstrated the presence of both the R1 and R2 subunits reliably in each airway, the latter as expected (Figure 5). We then examined whether these subunits were present in dAEC. Lysates from dAEC grown from six separate human airways were examined for the presence of R1 and R2 by Western blot. As shown in Figure 6A, the R2 subunit was expressed as expected, while R1 subunit protein expression was present in lower quantity. We then asked whether the combined IL-10 receptor was functional. To test this, we examined the phosphorylation of the potential downstream signaling protein STAT3 which is activated following IL-10 treatment in macrophages [53-55]. Cells were treated for up to 4 hr with 100 ng/ml of IL-10, after which whole cell lysates were resolved by Western blot. As shown in Figure 6B, there was no STAT3 phosphorylation. As IL-10 may also elicit phosphorylation of STAT1 [56,57], we examined phosphorylation of this transcription factor in the same lysates. No phosphorylation was seen (Figure 6B). Finally, we asked whether IL-10 treatment would elicit HLA-G5 expression. In these experiments, cells were treated for 24 hr with 1–100 ng/ml IL-10 or with 10,000 U/ml IFN-β as a positive control. As with treatment with either IL-4 or IL-13, there was no clear concentration response to IL-10 (Figure 6C), and the response to IL-10 was substantially less than that seen with IFN-β. These data suggested that the IL-10 receptor may not be functional in dAEC and that IL-10 treatment does not elicit HLA-G expression.

## Discussion

We demonstrate that HLA-G is expressed by differentiated airway epithelial cells in culture. Both G1 and G5 transcripts were demonstrated by qPCR, and soluble G5 and very modest membrane-bound G1 protein expression, were demonstrated by confocal microscopy. These data corroborate the finding of HLA-G in normal and asthmatic airways in vivo [22,26] and thus provide a useful model for studies of regulation and secretion. Further, we demonstrate that the Th2-associated cytokines IL-13, IL-4, and IL-5, and the immunoregulatory cytokine IL-10, do not regulate the expression of HLA-G in dAEC.

Epithelial cell secretion of HLA-G may have a role in regulating inflammation in asthmatic airways. Soluble isoforms of HLA-G secreted either into the airway lumen or into the local circulation may have a paracrine role in down-regulating inflammatory cells within airways, and in extending its influence beyond the epithelial layer where it can directly contact lymphocytes and dendritic cells in and near the mucosa. Although there is no direct evidence yet for this hypothesis in asthma, in other contexts HLA-G has been shown to suppress dendritic cells and T cells [27,33] that participate in inflammation, and to activate CD4+CD25+FoxP3+ regulatory T cells [58] that can suppress cells that participate in airway inflammation [59]. We have recently demonstrated increased presence of soluble HLA-G in bronchoalveolar lavage fluid recovered from subjects with mild asthma versus control, non-asthmatic subjects [26]; this may represent on-going attempts to suppress (incompletely or unsuccessfully) airway inflammation.

Because of this one could hypothesize that Th2-associated cytokines, demonstrated in increased abundance in asthmatic airways, would regulate HLA-G expression and abundance in airway epithelial cells that would in turn down-regulate certain aspects of airway inflammation would be counter-productive. The counter-hypothesis is that IL-4 and IL-13 drive airway inflammation and thus would not stimulate a counter-regulatory mediator such as HLA-G. The latter is correct: in our study, no Th2-associated cytokine stimulated HLA-G expression.

The overall role of Th2-associated cytokines in airway inflammation is clearly context-specific. Effector mechanisms by which these cytokines may defend the host appropriately (e.g., against infection) are the same that cause inappropriate or prolonged inflammation. Stimulation of AEC by IL-4 elicits production of several cytokines and chemokines [60-62] that contribute to airway inflammation in asthma, yet IL-4 also stimulates AEC migration, a pro-reparative process [40]. Stimulation of AEC by IL-13 elicits cytokine expression [61,62] as well as mucoid metaplasia [14,63], both of which are pro-inflammatory processes.

Our data also show that the IL-10R1 receptor subunit is expressed at low concentration in both airway epithelium in situ and in our dAEC culture model, and stand in contrast to the observations of Lim et al. [51]. However, despite the presence of both receptor subunits, treatment of dAEC with IL-10 did not elicit either STAT3 or STAT1 phosphorylation and thus, not surprisingly, we were unable to demonstrate significant HLA-G5 expression in response to this cytokine. Our data suggest that in contrast to expression seen in macrophages [64], IL-10 does not stimulate HLA-G expression in airway epithelial cells.

As our data are derived using an in vitro cell culture model, important influences on HLA-G expression may be absent, including local and circulating factors and the influence of cells beneath the basement membrane such as fibroblasts. Thus, the response to Th2-associated cytokines in the intact human airway may be different. However, the use of ALI culture permits examination of cytokine influences in isolation. Future experiments can examine the role of paracrine and circulating factors added to this system. HLA-G expression may also change as a function of disease state and inflammation in airways. Our experiments utilized cells collected from normal subjects, and it is possible that asthmatic epithelium may respond differently, either in a culture system or in the intact, inflamed airway. Careful comparison and correlation then will be required to understand specific receptor expression changes in a developing or repairing epithelium in vivo.

In conclusion, our study demonstrates the presence of HLA-G in differentiated airway epithelium in culture and its lack of regulation by Th2-associated cytokines such as IL-4 and IL-13 and by the immunomodulatory cytokine IL-10. While the role of HLA-G in airway inflammation and asthma remains to be defined, its presence, particularly in greater abundance in asthmatic airways (54) and in plasma of asthmatic subjects [24,25], suggests that epithelial cell expression may be of importance in airway inflammation.

## Abbreviations

ALI: Air liquid interface; β2m: β2-microglobulin; CK5: Cytokeratin 5; dAEC: Differentiated airway epithelial cells; GM-CSF: Granulocyte-macrophage colony stimulating factor; HLA-G: Human leukocyte antigen-G; ILT: Inhibitory receptor Ig-like transcript; IFN: Interferon; IL: Interleukin; LILR: Leukocyte Ig-like receptor; qPCR: Quantitative real-time reverse transcription polymerase chain reaction; SEM: Standard error of the mean; Th2: T helper 2.

## Competing interests

The authors declare that they have no competing interests.

## Authors’ contributions

SW developed the project, supervised completion of experiments, performed data analysis and wrote the manuscript. DL assisted with project development and performed initial qPCR experiments. TF assisted with the performance of IL-10 experiments. RS performed later qPCR experiments, cell culture, and all confocal microscopy imaging. BL and BM performed western blot experiments; BL also performed cell culture and supervised quality control. All authors read and approved the final manuscript.
